# Study on inclusion of probiotic, prebiotic and its combination in broiler diet and their effect on carcass characteristics and economics of commercial broilers

**DOI:** 10.14202/vetworld.2015.225-231

**Published:** 2015-02-25

**Authors:** M. A. Saiyed, R. S. Joshi, F. P. Savaliya, A. B. Patel, R. K. Mishra, N. J. Bhagora

**Affiliations:** 1 Veterinary Dispensary Tarapur, District Panchayat Anand, Gujarat, India; 2 Department of Animal Genetics & Breeding, Veterinary College, Anand Agricultural University, Anand, Gujarat, India; 3 Poultry Complex, Veterinary College, Anand Agricultural University, Anand, Gujarat, India

**Keywords:** broiler, carcass traits, European Performance Efficiency Index, prebiotic, probiotic, Return Over Feed Cost, symbiotic

## Abstract

**Background and Aim:**

In today era, broiler industry facing a problem of price hiking of feed of broiler, also in competitive era there should be lower feed cost, lower feed conversion ratio, low feed consumption yet good body weight at marketable age.

**Materials and Methods:**

Day-old commercial broiler chicks (n=200) were distributed randomly into 5 dietary treatment groups *viz*. control (T_1_), probiotic in the feed @ 100 g/tonne of feed (T_2_), prebiotic in the feed @ 500 g/tonne of feed (T_3_), probiotic + prebiotic @ 100 g/tonne and 500 g/tonne of feed, respectively (T_4_) and probiotic + prebiotic @ 50 g/tonne and 250 g/tonne of feed (T_5_). The growth of broilers and dressing weight along with the weight of giblet (liver without gall bladder, gizzard without serous layer, and heart without pericardium), Kidney, Abdominal fat, Length of Intestine and dressing percentage were measured. Economics in terms of Return Over Feed Cost (ROFC) and European Performance Efficiency Index (EPEI) was calculated.

**Results:**

Among all carcass traits, dressing percentage, abdominal fat weight and abdominal fat percentage (as a percentage of dressed weight) were recorded significant (p<0.05) difference among different treatment groups. The income from selling of the birds was significantly (p<0.05) higher in all treatment groups than the control group but there was a non-significant difference between supplemented groups. Feed cost during whole experimental period was significantly (p<0.05) lower in synbiotic supplemented groups (T_4_ and T_5_) than other groups. ROFC of all treatment group found significantly (p<0.05) higher than the control group.

**Conclusion:**

It can be concluded that the diet supplemented with synbiotic (100% level) was most efficient in terms of EPEI and synbiotic (50% level) in terms of ROFC. Hence, as feed supplement, synbiotic has a beneficial effect over probiotic and prebiotic when used alone.

## Introduction

Poultry is one of the fastest growing segments among the component of livestock sector in India. Production of agricultural crops has been growing at a rate of 1.5-2.0% per annum while poultry industry is growing at 8-15% per annum in India. The organized sector of the poultry is contributing nearly 70% of the total output and the rest 30% by the unorganized sector. About 66.7% of the total output from poultry is realized from the poultry meat sector and only 33.3% from egg production. The total poultry population, which was only 73.5 million in 1951 has made tremendous growth during the past 50 years and has reached 648.83 million as per 2007 Census. India occupied the 3^rd^ position in egg production and 5^th^ position in poultry meat production as per 2010 census in the world. The per capita availability of poultry meat is 2.15 kg/annum, which is very less as against the recommendation of 11 kg meat/annum given by National Institute of Nutrition [1].

Feed as a major input item to broiler rearing for being 75% of the production cost has a vital role in broiler economics. Hence, it is imperative to give due attention to proper utilization of feed without adversely affecting the growth or production performance of broilers [2]. Antibiotics have been also used to promote growth rate, improve feed conversion ratio (FCR) and reduce mortality in broiler flocks. However, repeated use of antibiotics in poultry diets resulted in severe problems like resistance of pathogen to antibiotics, accumulation of antibiotics residue in their products and environment, imbalance of normal microflora and reduction in beneficial intestinal microflora [3,4].

This has led to the development of different products to be used as feed additives such as enzymes, probiotics, prebiotics, organic acids, and plant extracts. Probiotics are “live microorganisms, which when administered in adequate amounts confer a health benefit on the host” [5]. Whereas prebiotics are defined as non-digestible food ingredients that beneficially affect the host by selectively stimulating the growth and activity of one or a limited number of bacteria. Synbiotics refer to nutritional supplements combining probiotics and prebiotics and in a form of synergism. The main reason, for using a synbiotic, is that a true probiotic, without its prebiotic food, does not survive well in the digestive system. Without the necessary food source for the probiotic, it will have a greater intolerance for oxygen, low pH, and temperature [6]. Poultry meat production has been paid more and more attention because it is particularly high in quantities and qualities of valuable protein, essential amino acids, fat, essential fatty acids, vitamins and minerals. Poultry meat is a high quality concentrated food and, therefore, plays an important role in human nutrition [7]. Poultry meat constitutes around 20% of total meat production in the country [8].

## Materials and Methods

### Ethical approval

The present study was carried out at the age of 42 days/6 week and carcass parameters were carried out at chicken shop with due care after selling of the birds.

### Experimental design

A 200-day-old commercial broiler chicks were randomly distributed into five different treatment groups having four replicates in each treatment with 10 chicks in each replicate and they were reared under battery brooder system of cage up to 42 days of age.

### Experimental procedure

Feed and water were offered *ad libitum*, and standard managemental practices followed. Chicks were weighed individually at the start of the experiment and at the end of every week. Mortality was also recorded. Composition of dietary treatments is given in Table-1. Proportion of different feed ingredient is shown in Table-2 and detailed composition of feed additives is shown in Table-2.1 First group of birds were kept as a control, and they were not supplied feed with either probiotic or prebiotic in both, broiler starter and broiler finisher feed. Probiotic in the feed of T_2_ group was given at the rate of 100 g/tonne of feed during the starter phase (0-4 weeks) and finisher phase (5-6 weeks). Prebiotic in the feed of T_3_ group was given at the rate of 500 g/tonne of feed during the starter phase and finisher phase. In T_4_ group, probiotic was given at the rate of 100 g/tonne of feed and Prebiotic was given at the rate of 500 g/tonne of feed during the starter phase and finisher phase. Whereas, in T_5_ group, probiotic was given at the rate of 50 g/tonne of feed and prebiotic was given at the rate of 250 g/tonne of feed during starter phase and finisher phase i.e. half of the dose than T_4_ treatment. Each gram of probiotic contains 6 × 10^9^ colony forming units of *Lactobacillus acidophilus, Lactobacillus casei, Pediococcus acidilactici, Bacillus subtilis* and *Saccharomyces boulardii*. Whereas, prebiotic contains Mannan Oligo-Saccharide in which Mannan 12-14% and Glucan 13-16% were included. The broiler starter (0-28 days) and broiler finisher (29-42 days) feeds for different treatments were prepared as per the guidelines of BIS (1992). Return Over Feed Cost (ROFC) is calculated by subtracting the feed cost during the rearing period from the income from the sold bird on live weight basis. Whereas European Performance Efficiency Index (EPEI) (European Performance Efficiency Index), as described by [9], was calculated by following formula.

**Table-1:** Composition of dietary treatment employed.

Dietary treatment	Composition
T_1_	Basal diet without additive, served as control
T_2_	Basal diet+Probiotic @ 100 g/tonne of feed
T_3_	Basal diet+Prebiotic @ 500 g/tonne of feed
T_4_	Basal diet+Probiotic @ 100 g/tonne of feed+Prebiotic @ 500 g/tonne of feed
T_5_	Basal diet+Probiotic @ 50 g/tonne of feed+Prebiotic @ 250 g/tonne of feed

**Table-2:** Proportion of feed ingredients (%) used for preparation of broiler starter and finisher feeds.

Ingredients	Proportion (Kg)	

	Broiler starter	Broiler finisher
Maize	53.900	54.075
Deoiled rice bran	1.150	9.150
Soyabean DOC	39.755	30.500
Trace mineral^1^	0.100	0.000
Shell grit	2.200	1.700
DCP	2.000	1.390
Salt	0.300	0.400
Enzymes^2^	0.050	0.050
Furazolidone^3^	0.025	0.050
Metabolic activator^4^	0.100	0.100
Toxin binder^5^	0.100	0.100
Maduramicin^6^	0.050	0.050
Lysine	0.100	0.100
DLMethionine	0.135	0.090
Herbal performance enhancer^7^	0.025	0.025
Vitamin B_12_^8^	0.010	0.020
Vegetable oil	0.000	2.000
Vitamin and mineral supplement^9^	0.000	0.200
Total	100.00	100.00
CP (%)*	23.10	20.20
ME (kcal/kg feed)*	2800.00	2900.00

* As per calculated values, DCP=Digestible crude protein

**Table-2.1:** Detailed composition of feed additives added in feed.

1. Trace Minerals:
Each kg contains:
Copper-15 g, Iodine-1 g, Iron-60 g, Manganese-80 g, Selenium-0.3 g, Zinc-80 g, Inorganic nutritive care-Q.S.
2. Enzymes:
Each gram contains:
Xylanase-2000 IU, Amylase-400 IU, Protease-4000 IU, Cellulase-500 IU.
3. Furazolidone:
Each kg contains:
Furazolidone-200 g, Inorganic nutritive carrier-Q.S.
4. Metabolic activator:
Lecithin extract treated with co enzyme.
5. Toxin binder:
Selected silicates, surfactants, organic acids and salts of organic acids.
6. Maduramicin:
Ionophore (polyether antibiotic) coccidiostat.
7. Herbal performance enhancer @ 250 g per tonne of feed.
8. Vitamin B_12_:
Each kg contains: Vitamin B_12_-100 mg
9. Vitamin and mineral supplement:
Each 2 kg contains:
Vitamin A-50 lakh IU, Vitamin B2-2 g, Vitamin B6-400 mg, Vitamin B12-5600 mcg, Vitamin E-800 IU, Iron-7.5 g, Vitamin D3-6.25 lakh IU, Choline chloride-10 g, Copper-2 g, Iodine-1 g, Zinc-15 g, Manganese-27.5 g, Calcium-27.25%, Phosphorus-7.45%, Calcium pantothenate-4 g.


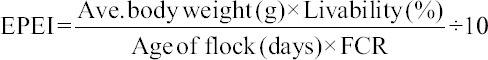


### Statistical analysis

The data on various traits were analyzed using completely randomized design as per Snedecor and Cochran (1995) [10].

### Results and Discussion

The carcass traits like dressed weight, dressing%, liver weight, heart weight, gizzard weight, giblet weight, giblet%, abdominal fat weight, abdominal fat%, intestinal length and kidney weight of the different groups offered feed additives either singly or in combination in relation to control is presented in Table-3.

**Table-3:** Carcass characteristics of commercial broilers fed with or without probiotic, prebiotic or synbiotic supplemented feed.

Carcass trait	Treatments					CD value

	T_1_	T_2_	T_3_	T_4_	T_5_	
Pre-slaughter weight (g)	1965.00±46.94	1950.00±20.41	1932.50±34.97	1945.00±35.70	1925.00±14.43	NS
Dressed weight (g)	1247.75±31.65	1312.75±15.19	1309.50±30.80	1305.00±42.28	1326.50±10.74	NS
Dressing %	63.51^a^±0.99	67.35^b^±1.06	67.76^b^±1.01	67.06^b^±1.24	68.92^b^±0.64	3.04
Liver weight (g)	40.75±2.21	42.13±1.08	40.50±1.32	45.25±1.70	46.00±2.16	NS
Heart weight (g)	8.75±0.52	9.50±0.64	7.75±0.85	8.75±0.47	7.88±1.08	NS
Gizzard weight (g)	34.25±1.65	36.38±3.41	37.25±0.62	32.75±3.68	34.38±2.62	NS
Giblet weight (g)	83.75±2.77	88.00±4.14	85.50±0.95	86.75±4.49	88.25±3.78	NS
Giblet %	6.72±0.25	6.70±0.30	6.54±0.11	6.64±0.21	6.65±0.25	NS
Abdominal fat weight (g)	38.63^b^±1.65	32.50^a^±1.50	36.00^ab^±1.22	31.75^a^±1.18	33.75^a^±1.75	4.45
Abdominal fat %	3.10^b^±0.15	2.47^a^±0.09	2.76^ab^±0.13	2.45^a^±0.16	2.54^a^±0.11	0.41
Intestinal length (inches)	79.50±2.59	82.25±1.93	78.00±3.34	80.25±0.85	81.00±2.04	NS
Kidney weight (g)	15.75±1.31	14.00±2.04	15.00±1.87	14.25±1.31	16.50±1.89	NS

*Means within row with different superscript differ significantly (p<0.05), NS=Non-significant

### Dressed weight

The average body weight gain for different treatment group and for weekly interval as well as for starter and finisher phase is given in Table-4. The highest dressed weight was observed in synbiotic half level group (T_5_), which was followed by T_2_, T_3_, T_4_ and T_1_ groups. Dressed weight was highest in T_5_ though it had the lowest pre-slaughter weight. The dressed weight differences were non-significant amongst all treatment groups. Present findings were in accordance with [11-14].

**Table-4:** Average body weight gain (g) of commercial broilers under different treatments for weekly interval up to 6 weeks age.

Weeks	Treatments					CD value

	T_1_	T_2_	T_3_	T_4_	T_5_	
0-1	81.53^a^±1.96	110.10^b^±3.62	107.76^b^±1.89	103.74^b^±3.06	104.45^b^±4.45	9.51
1-2	133.98±4.62	152.73±3.90	155.94±4.86	150.91±5.00	155.88±8.19	NS
2-3	296.89±8.15	296.63±9.44	312.30±9.60	313.40±13.05	306.95±9.02	NS
3-4	438.35±10.40	455.50±6.88	461.03±10.84	446.64±12.24	488.32±39.12	NS
4-5	473.18±21.74	477.45±16.47	481.33±9.90	515.35±7.23	488.89±8.68	NS
5-6	489.23±5.21	517.30±28.45	535.58±4.00	499.40±21.42	499.00±17.37	NS
0-4	950.74±12.33	1014.94±17.39	1037.03±12.28	1014.69±30.19	1055.59±39.98	NS
4-6	962.40±25.23	994.75±23.83	1016.90±7.15	1014.75±24.45	987.89±24.60	NS
0-6	1913.14±31.87	2009.69±36.74	2053.93±14.25	2029.44±28.11	2043.49±58.34	NS

*Means within row with different superscript differ significantly (p<0.05)

### Dressing yield (%)

**Figure-1:**
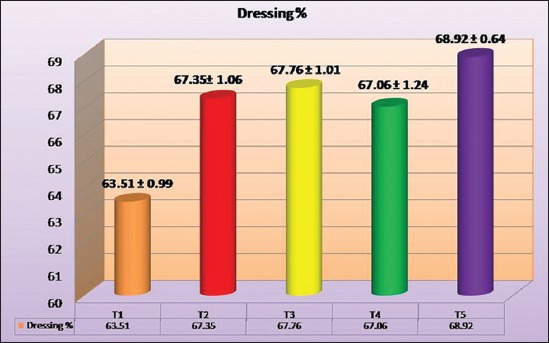
The average dressing percentages of birds under different treatments.

The highest dressing per cent was observed in synbiotic half level group (T_5_) which was followed by T_3_, T_2_, T_4_ and T_1_ group and all treatment groups dressing percentages were significantly (p<0.05) higher than control group but with non-significant difference between supplemental groups. Present findings differed from [11-13,15,16] as they all observed non-significant differences between supplemental groups and control groups. The average dressing percentage for different treatment groups is shown in Figure-1.

### Liver weight

The highest liver weight was observed in synbiotic half level group (T_5_), which was followed by T_4_, T_2_, T_1_ and T_3_ group. There were a non-significant difference between all the treatment groups for liver weight [13,14,17,18] found similar findings.

### Heart weight

The heart weight with the highest value observed in the probiotic group (T_2_) which was followed by T_1_, T_4_, T_5_ and T_3_ group. There was non-significant difference between all the treatment groups [13,14,17] found similar findings to this study.

### Gizzard weight

**Figure-2:**
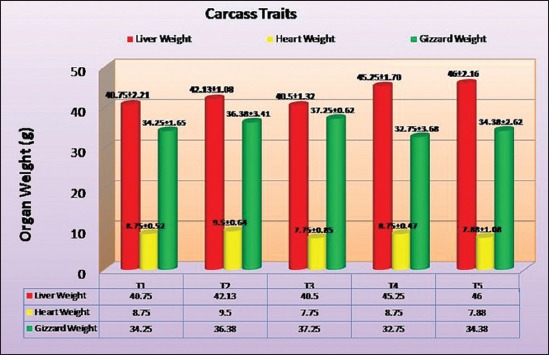
The average weight of organs included in giblet under different treatment groups.

The highest gizzard weight was observed in the prebiotic group (T_3_) which was followed by T_2_, T_5_, T_1_ and T_4_ group. There was non-significant difference between all the treatment groups [13,17,18] also reported similar findings. The average weight of the organs has been shown in Figure-2.

### Giblet weight

The highest giblet weight was observed in synbiotic half level group (T_5_) which was followed by T_2_, T_4_, T_3_ and T_1_ group. There were non-significant differences between all the treatment groups.

### Abdominal fat weight and %

The control group (T_1_) birds were having highest abdominal fat % which was significantly (p<0.05) higher than T_5_, T_2_ and T_4_ group though there was a non-significant difference between control and prebiotic supplemented group (T_3_). The lowest abdominal fat % was observed in the synbiotic group (T_4_). The highest abdominal fat % in the control group was followed by T_3_, T_5_, T_2_ and T_4_ groups. Present study was in accordance with results of [11,14,19]. Present study differed from results of [12,15,16,20].

### Intestinal length

The highest intestinal length was found in the probiotic supplemented group (T_2_) which was followed by T_5_, T_4_, T_1_ and T_3_. The intestinal length was not differing among each other though numerical differences were there. Present study was in accordance with findings of [16] but values for intestinal length were lower than present findings.

### Kidney weight

The highest kidney weight was noticed in the synbiotic half level group (T_5_) which was followed by T_1_, T_3_, T_4_ and T_2_ group and there were non-significant differences amongst each other. Present study was in accordance with the results of [14] who observed lower kidney weight than the present study.

Amongst all carcass traits, dressing percentage, abdominal fat weight and abdominal fat percentage (as a percentage of dressed weight) were recorded significant (p<0.05) difference amongst different treatment groups. Synbiotic half level supplemented (T_5_) was dominating for most of the traits. Higher dressed weight, higher dressing percentage, higher liver weight, higher giblet weight and higher kidney weight was observed in synbiotic half level supplemented group (T_5_) than all other treatments. Synbiotic group (T_4_) was having lower abdominal fat weight and lower abdominal fat percentage (as a percentage of dressed weight) among all treatment. Prebiotic group (T_3_) had higher gizzard weight than other groups. Whereas, a probiotic group (T_2_) had higher heart weight and longer intestinal length amongst all supplemental groups. Giblet percentage (as a percentage of dressed weight) was higher in the control group.

### ROFC

**Figure-3:**
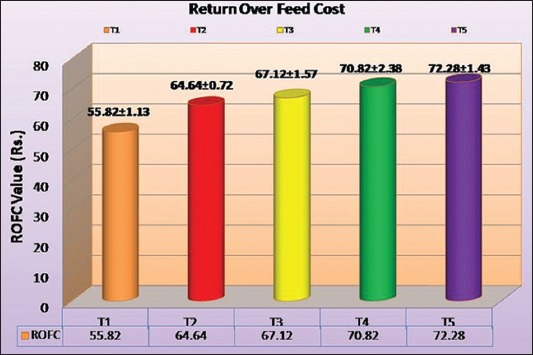
The Return Over Feed Cost (ROFC) for various treatment groups in commercial broilers.

Average ROFC in terms of (Rs./bird), (%/bird), income from selling and feed cost of broilers under different treatment groups has been shown in Table-5 and Figure-3. Cost of feed per kg for different treatment groups is shown in Table-6. ROFC in terms of % per bird was indicating the per cent of profit in treatment as compared to the control group. Average daily feed consumption (g/bird/day) is given in Table-7. The income from selling of the birds was significantly (p<0.05) higher in all treatment groups than the control group but there was a non-significant difference between supplemented groups. Feed cost during whole experimental period was significantly (p<0.05) lower in synbiotic supplemented groups (T_4_ and T_5_) than other groups. ROFC of the control group found significantly (p<0.05) lower than all treatment groups. Non-significant difference was found between T_2_ and T_3_, T_3_ and T_4_ and T_4_ and T_5_. In terms of percentage, highest ROFC was in T_5_ (29.48%) than T_4_ (26.87%) followed by T_3_ (20.24%) and T_2_ (15.80%) as compared to control (T_1_) suggesting incorporation of synbiotic yields more return and we can afford its 50% level for better economy. The present study was in accordance with [11,21,22] though they observed lower values of profit as compared to present findings, but the trend was same.

**Table-5:** The ROFC (Rs/bird) and (%/bird) realized under different feed supplement groups.

Particulars	Treatment					CD value

	T_1_	T_2_	T_3_	T_4_	T_5_	
Income from bird sold (Rs./bird)	141.32^a^±2.25	148.28^b^±2.62	151.44^b^±1.01	149.67^b^±2.03	150.75^b^±1.31	5.85
Feed cost (Rs./bird)	85.50^b^±1.34	83.63^b^±2.38	84.32^b^±0.60	78.85^a^±1.14	78.47^a^±1.18	4.37
ROFC (Rs./bird)	55.82^a^±1.13	64.64^b^±0.72	67.12^bc^±1.57	70.82^cd^±2.38	72.28^d^±1.43	4.66
ROFC (Rs./kg live weight)	28.43	31.38	31.91	34.06	34.52	-
ROFC (%/bird)	00.00	15.80	20.24	26.87	29.48	-

ROFC=Return over feed cost, *Means within row with different superscript differ significantly (p<0.05)

**Table-6:** Cost of feed of control and supplemented with probiotic, prebiotic and synbiotic.

Type of feed	T_1_	T_2_	T_3_	T_4_	T_5_
Broiler starter (Rs./kg)	22.32	22.49	22.39	22.56	22.44
Broiler finisher (Rs./kg)	22.02	22.19	22.09	22.26	22.14

**Table-7:** Average daily feed consumption (g/bird/day) of commercial broilers for different treatments during different time intervals.

Weeks	Treatments					CD value

	T_1_	T_2_	T_3_	T_4_	T_5_	
0-1	20.13±0.14	19.31±0.18	19.38±0.20	19.50±0.35	19.16±0.56	NS
1-2	41.58^b^±0.50	36.83^a^±0.12	36.64^a^±0.17	37.08^a^±0.30	36.86^a^±0.15	0.86
2-3	67.84±0.84	69.92±1.03	69.60±0.43	69.37±0.59	68.59±0.68	NS
3-4	133.26^c^±1.26	126.70^c^±4.78	129.34^c^±2.47	115.48^b^±1.57	107.13^a^±2.04	8.23
4-5	152.77±4.34	137.70±6.25	136.33±2.00	137.79±4.10	136.75±2.31	NS
5-6	135.54^ab^±3.95	144.56^bc^±6.50	150.55^c^±0.98	123.58^a^±5.34	134.73^ab^±3.66	13.54
0-4	65.70^d^±0.51	63.19^c^±1.09	63.74^cd^±0.67	60.36^b^±0.52	57.93^a^±0.58	2.14
4-6	144.16±3.95	141.13±5.58	143.44±1.03	130.68±2.97	135.74±2.83	NS
0-6	91.85^b^±1.44	89.17^b^±2.54	90.31^b^±0.64	83.80^a^±1.21	83.87^a^±1.26	4.67

*Means within row with different superscript differ significantly (p<0.05)

### EPEI

**Figure-4:**
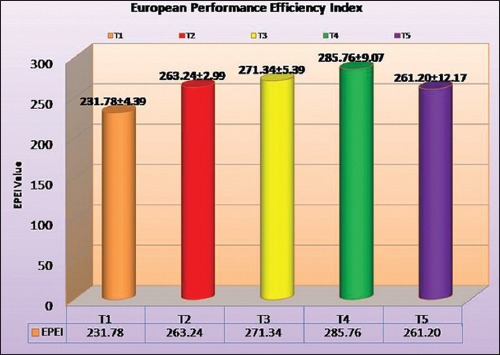
European Performance Efficiency Index (EPEI) for various treatment groups in commercial broilers.

The EPEI for performance of the broilers is given in Table-8 and Figure-4. The EPEI value was higher in T_4_ (285.76) which was followed by T_3_ (271.34), T_2_ (263.24), T_5_ (261.20) and T_1_ (231.78). All supplemented groups were having significantly (p<0.05) higher EPEI value than the control. There was a non-significant difference between T_2_, T_3_, T_5_ and T_2_, T_3_, T_4_. The higher EPEI value means higher average body weight; good livability and higher feed efficiency in stipulated number of days thus give overall economics of the birds considering various important traits. Hence, T_4_ group (synbiotic) found more economical than other group when EPEI considered.

**Table-8:** The EPEI of broilers fed with Probiotic, Prebiotic and their combination under different treatment.

Particulars	Treatment					CD value

	T_1_	T_2_	T_3_	T_4_	T_5_	
EPEI	231.78^a^±4.39	263.24^bc^±2.99	271.34^bc^±5.39	285.76^c^±9.07	261.20^b^±12.17	22.87

EPEI=European performance efficiency index, *Means within row with different superscript differ significantly (p<0.05)

## Conclusion

Dressing percentage was highest in synbiotic (50% level) group (T_5_) over other supplemented groups and differed significantly with control in spite of having lowest pre-slaughter weight. The ROFC (Rs.) was significantly (p<0.05) higher in the synbiotic group (T_4_ and T_5_) than other groups. Also, ROFC in terms of per cent per bird was highest in synbiotic (50% level) group (T_5_) than all other groups. EPEI was highest in symbiotic (100%) group (T_4_) over other group which means higher average body weight; good livability and higher feed efficiency in stipulated number of days by the group.

Overall it can be concluded that the diet supplemented with synbiotic (100% level) was most efficient in terms of EPEI and synbiotic (50% level) in terms of ROFC. Hence, As feed supplement, synbiotic has a beneficial effect over probiotic and prebiotic when used alone.

## Authors’ Contributions

RSJ designed the experiment. MAS, FPS and RSJ carried out the research. MAS, ABP and RKM drafted and revised the manuscript. ABP, NJB, RSJ and FPS recorded and analysed the data All authors read and approved the final manuscript.
